# One-Year Results of the *BeweegKuur* Lifestyle Intervention Implemented in Dutch Primary Healthcare Settings

**DOI:** 10.1155/2015/484823

**Published:** 2015-08-25

**Authors:** Bianca A. M. Schutte, Annemien Haveman-Nies, Liesbeth Preller

**Affiliations:** ^1^Division of Human Nutrition, Wageningen University, Bomenweg 4, 6703 HD Wageningen, Netherlands; ^2^Netherlands Institute for Sport and Physical Activity, Horapark 4, 6717 LZ Ede, Netherlands

## Abstract

*Background*. Lifestyle interventions focusing on healthy diet and physical activity (PA) are effective in reducing health risks in controlled research settings. The aim of this study was to investigate the one-year results of the *BeweegKuur* lifestyle intervention implemented nationwide in Netherlands for people with a weight-related health risk. *Materials and Methods*. Data were requested from all 160 locations participating in the *BeweegKuur*. In a one group pretest/posttest study, one-year changes in health outcome variables and time spent on physical activity were tested with dependent *t*-tests. Associations between one-year changes in weight and waist circumference and sociodemographic factors and uptake of the program were analysed with ANOVA. *Results*. Data for 517 participants from 47 locations were available for analysis. One year after the intervention, weight reduced by 2.9 kg (95% CI −3.3;, −2.5), waist circumference by 4.3 cm (−4.9; −3.7), and blood glucose by 0.5 mmol/L (−0.8; −0.3). Physical activity increased significantly. Higher uptake of the program was associated with a larger decrease in waist circumference. *Conclusion*. The results of the study suggest that lifestyle interventions implemented in real-life primary healthcare settings with tailor-made supervision can contribute meaningfully to primary prevention.

## 1. Introduction

Worldwide, age-standardized prevalence of obesity in adults almost doubled between 1980 and 2008 [1]. Obesity is a significant risk factor for increased morbidity and mortality, most importantly for cardiovascular disease and type 2 diabetes mellitus [2, 3]. As inactivity and an unhealthy diet are very strong determinants of obesity, cardiovascular disease, and type 2 diabetes, much of the disease burden can be prevented or at least be postponed [4–6]. Lifestyle interventions combining a focus on sustainable change in diet, physical activity (PA), and behavioural change demonstrate promising results in reducing the risk of obesity and type 2 diabetes [7–9]. However, most lifestyle interventions aimed at reducing diabetes and cardiovascular risks are developed and tested in controlled research settings. They are usually intensive, highly standardized, and delivered by specially educated staff using strict protocols [10]. Different studies have been performed to determine whether results obtained in research settings could be replicated in real-life primary healthcare [11–15]. These interventions are implemented by regular primary healthcare professionals (general practitioner, practice nurse, dietician, and/or physiotherapist), and participants are supervised for a shorter intervention period than in research, of about one year. Overall, effects of these real-life interventions yield smaller effect sizes for health outcome variables compared to interventions in controlled research settings, or no effects are found [16].

Within a nationwide implementation project commissioned by the Dutch Ministry of Health, Welfare, and Sport, the* BeweegKuur* was implemented in local, real-life primary healthcare settings spread over Netherlands [17]. The objective of this study is to evaluate the effect of this lifestyle intervention on weight, waist circumference, and PA. In addition, the association between change in weight and waist circumference and level of uptake of the program and participants' sociodemographic characteristics is studied.

## 2. Materials and Methods

### 2.1. The* BeweegKuur* Lifestyle Intervention

The pilot* BeweegKuur* project was started in 2007 because the former Dutch Minister of Health wanted to implement a lifestyle intervention nationwide, accessible, and reimbursed by health insurance for the whole target group. Together with national stakeholders, an intervention was designed based on existing interventions and known effective elements. The intervention took place in the primary healthcare setting. The aim of the project was to optimize nationwide implementation [17]. This was accompanied by process evaluations [18, 19]. On the basis of these results, individual locations were allowed to adjust the* BeweegKuur* to a certain extent to the local situation. Also, these locations were allowed to use their own quality-assured procedures for testing and measuring health parameters. Locations applied voluntarily to join, resulting in 160 participating locations spread over Netherlands in 2011. Every location was allowed to include a maximum of 40 participants per year. Local healthcare professionals were supported by one of 20 regional primary care support structures, which in turn were instructed by the Netherlands Institute for Sport and Physical Activity (NISB). All professionals supporting participants received training on the* BeweegKuur*; lifestyle advisors (LSAs) and physiotherapists received a three-day course and the dieticians a one-day course.* BeweegKuur* protocols and other guidelines were available for all healthcare professionals.

The aim of the* BeweegKuur* intervention in itself was to achieve health benefits through increased PA and improved dietary behaviour. The* BeweegKuur*'s starting point was the general practitioner's (GP) practice. The GP referred patients with a weight-related health risk (see Section 2.2 for inclusion criteria) to an intake session with an LSA, commonly a practice nurse. Shortly afterwards, the exact time depending on the location, a physiotherapist performed an endurance test. The LSA used this test to assign the participant to exercise programs 1, 2, or 3, varying in intensity of guidance by the LSA and physiotherapist (Table 1). Throughout the year, participants had around seven tailor-made coaching and supervision sessions with the LSA based on principles of motivational interviewing [20] and the self-determination theory [21]. In addition, all participants were referred to a dietician. Participants could start with the intervention whenever it suited them, in consultation with the LSA, but the duration of the intervention was one year for all participants. To increase PA and to contribute to sustainable changes in PA, participants were coached to increase PA in daily life and referred to local exercise facilities during the intervention. Again, participants had flexibility about when they could start at these facilities; the timing depended on the ability of the participant to exercise individually. The timing and type of all activities were highly tailored to the participant and designed in close consultation with the participant and the physiotherapist. The number of consultations with the LSA, dietician, and physiotherapist was higher in the beginning of the intervention and decreased gradually during the year.

### 2.2. Study Design and Participants

In this study, a one group pretest/posttest study design was used. Data were collected per location by the LSA and entered in a standardized registration file, administered by NISB. In autumn 2011, these registration files were requested for this study from the LSAs in all 160 locations. The majority of participants started with the intervention in 2009 and 2010.

The GP was responsible for screening for contraindications, based on current medical guidelines and standards in the Netherlands, and decided whether a person could participate in the* BeweegKuur*. Prospective participants had to meet certain inclusion criteria. They were included if they

- were motivated to change behaviour;
- did not meet the Dutch Standard for Healthy Exercise (at least 30 minutes of moderate physical activity on at least five days a week);
- had a BMI between 25 and 30 in combination with a waist circumference ≥88 cm for women and ≥102 cm for men and/or comorbidity;
- had a BMI between 30 and 35, regardless of waist circumference and comorbidity;
- had a BMI between 35 and 40, regardless of waist circumference but without comorbidity.

Comorbidity included hypertension, dyslipidaemia, diabetes mellitus, cardiovascular disease, osteoarthritis, and sleep apnoea.

### 2.3. Measurements

Measurements were taken during sessions with the LSA and included weight, BMI, waist circumference, blood glucose, and blood pressure. Data from the first and last sessions were used for the analyses. Anthropometric and blood pressure measurements were conducted according to standardized procedures in the GP practice. For blood glucose, LSAs registered whether blood glucose was measured in a fasting state or not, according to local procedures. Only fasting-state measurements were used for analysis. Physical activity was self-reported using the standardized short version of the validated SQUASH questionnaire [22]. Activities performed at work, during household activities, leisure time, and commuting were classified into the categories light to moderate and vigorous on the basis of their intensity. Sociodemographic factors like age, gender, smoking behaviour, and educational level were recorded by the LSA using standardized questionnaires. To assess uptake of the program, the number of sessions with the dietician and the LSA and attendance at the group education lessons with the dietician were recorded by the LSA.

### 2.4. Statistical Analysis

Data analyses were conducted using SPSS for Windows (version 18). One-year changes in weight, BMI, waist circumference, blood glucose, blood pressure, and physical activity were normally distributed for the overall population and the different subgroups. One-year changes were tested with a paired samples' *t*-test. A *p* value of <0.05 was considered to be statistically significant. All tests were two-sided. ANOVA was conducted to test whether sociodemographic factors, uptake of the program, and change in physical activity were related to changes in weight and waist circumference, followed by the Games-Howell post hoc test (*p* < 0.05) [23].

## 3. Results

The LSAs from 81 of the 160 locations sent back the registration files. Complete data for 517 participants from 47 locations (mean = 11 persons/location) were available for analysis; participants in the other locations had not yet finished the intervention at the time of data retrieval.

The background characteristics of participants are described in Table 2. Mean age was 58 years; most participants were between 50 and 70 years old. The majority of participants were female (59.2%). Most participants had low (39.5%) or intermediate (44.4%) levels of education. Compared to the Dutch population of the same age, participants were less educated and more often married (Supplementary Table 1 in Supplementary Material available online at http://dx.doi.org/10.1155/2015/484823).

One year after the start of the intervention, weight and waist circumference had significantly reduced by 2.9 kg (3.0% of baseline) and 4.3 cm (3.8%) (Table 3). Other health parameters showed the same trend. Light to moderate PA and vigorous PA increased by 2.1 (15%) and 1.7 (40%) hours a week, respectively. Males and females differed in the anthropometric outcomes and PA at baseline, but the effects on weight and waist circumference were similar for men and women (Supplementary Table 2).

Younger participants lost on average more weight than older participants (Table 4). Larger reductions in weight and waist circumference were seen in participants in the highest BMI category. Generally, larger changes in waist circumference were associated with a higher uptake of the program: waist circumference decreased more in participants with more supervision from the physiotherapist and six or more sessions with the LSA.

## 4. Discussion

This study has shown that participation in the real-life lifestyle intervention* BeweegKuur* was positively related to participants reducing weight, waist circumference, blood pressure, and blood glucose and increasing their physical activity. The largest changes in waist circumference were observed in participants with higher uptake of the program.

In this study, weight and waist circumference reduced by 2.9 kg and 4.3 cm, respectively, after one year. These effects were stronger than that found in a previous effect evaluation of an earlier and less developed version of the* BeweegKuur* carried out in a few locations [24]. The larger effects in this study may be caused by an increase in the professional development of LSAs, physiotherapists, and dieticians as well as the improved attention given to PA outside the intervention. Also, in comparison with other lifestyle interventions implemented in practice, that is, Australian Greater Green Triangle (GGT) Diabetes Prevention Project [12], the Dutch APHRODITE study [15], the Finnish GOAL Intervention Study [11], and the Finnish National Diabetes Prevention Program (FIN-D2D) [14], the* BeweegKuur* appeared to be more effective. In these interventions, weight loss was on average between 0.5 and 2.5 kg and waist circumference decreased from 0.4 to 4.2 cm after 1 to 1.5 years.

In the* BeweegKuur*, participants were referred to the most appropriate exercise program, depending on the level of weight-related health risk. Participants followed sessions with the physiotherapist, and the LSA designed a tailor-made program and provided coaching and supervision, according to the person's needs. Additionally, participants were referred to local sport facilities with personalized programs adjusted to their physical and mental capabilities. In none of the other intervention studies participants received similar tailor-made supervision to increase their PA [11, 12, 14, 15, 25]. The increase in PA was also larger in the* BeweegKuur* compared with other intervention studies. In the* BeweegKuur*, light to moderate and vigorous PA were increased by 2.1 and 1.7 hours a week, respectively. The Hoorn Prevention Study found a decrease in PA after one year [13] and the APHRODITE study found an increase in PA after half a year and a decrease after 1.5 and 2.5 years [15, 26]. The considerable effect of the* BeweegKuur* on PA may be due, at least partly, to the strong focus on optimal implementation, based on the results of extensive formative evaluation in relation to the nationwide implementation project [18, 19].

In the* BeweegKuur*, participants had individual sessions with the LSA, but most sessions with the dietician and the physiotherapist were in groups. Alongside the benefits of individual coaching, group counselling can promote group cohesion, generally having a beneficial effect on behaviour change [17, 19, 27–29]. In other interventions implemented in practice, most sessions with healthcare professionals were individual, or the number of group sessions was minimal [14, 25]. Moreover, the* BeweegKuur* was an extensive program, and uptake of the program was high in comparison with other interventions. Fifty-five percent of* BeweegKuur* participants visited the LSA six times or more. In the FIN-D2D program, only 29% of the participants had three or more sessions in usual primary healthcare [14], and 43% and 57% of the participants attended a maximum of six counselling sessions in the GGT Diabetes Prevention Project and the GOAL intervention, respectively [11, 12]. We found stronger effects on waist circumference in persons who had more consultations with primary healthcare professionals and attended group sessions.

Initially, the* BeweegKuur* was designed as an implementation project aimed at optimizing local and nationwide implementation of lifestyle interventions and not primarily as a research project [17]. Consequently, the* BeweegKuur* was well embedded in local-practice working standards, but the structure of the project led to several drawbacks for this study. No control group could be included and the response rate was relatively low, thereby limiting adjustment for potential confounders.

The design of the whole project did not include very strict quality control procedures for data collection. Individual LSAs were responsible for data entering. This might have led to potential information bias, with structural overestimation of the results of the intervention. Differences in measurement methods could also have led to bias but are unlikely to contribute to overestimation of associations. All measurements were carried out with instruments and tests available in professional general practices. These instruments meet professional quality standards, and therefore we believe that the effect on measured differences is within the range of total variation of these parameters.

The study locations included in the analysis were spread across the country, representing the Dutch target group. The study population might be a selective group, as data for persons who did not complete the* BeweegKuur* were not collected. In Vermunt et al.'s study [30] on the effectiveness of the Dutch APHRODITE study, a lifestyle intervention in primary healthcare, it was observed that dropouts had similar clinical outcomes (body weight, blood glucose values) on baseline as completers.

Notwithstanding these methodological limitations, this study can contribute to a growing understanding of an effective implementation methodology regarding lifestyle interventions in real-life primary care settings, as knowledge on this essential step is limited. This study gives an indication that the essential elements of the intervention seem to be a number of sessions with the healthcare professionals, a combination of individual and group sessions, tailor-made supervision and counselling, and referral to local sport facilities with personalized programs. The latter element is described in detail by Elsman et al. [29].

## 5. Conclusion

One-year results of the* BeweegKuur* lifestyle intervention demonstrated positive results on physical activity and anthropometric outcomes. Due to limitations of the study design, it cannot be ruled out that reported outcomes overestimate the results that can be achieved in the entire population. However, the effect evaluation indicates that a well-implemented intervention, combining individual and group sessions and tailor-made supervision by local healthcare professionals, can result in substantial lifestyle and health changes in persons who fully participate.

## Supplementary Material

Supplemental Table 1: shows the socio-demographic characteristics of the Dutch population and BeweegKuur intervention participants.
Supplemental Table 2: shows baseline measurements and changes in anthropometric outcomes and physical activity after one year split by gender.

## Figures and Tables

**Table 1:** Number of consultations with the different healthcare professionals per exercise program.

	Program 1 Independent exercise program^1^	Program 2 Start-up program^1^	Program 3 Supervised exercise program^1^
Lifestyle advisor	8	7	7
Physiotherapist	0	5	24–51^*∗*^
Individual sessions dietician	4	4	4
Group sessions dietician	7	7	7

^1^Number of consultations was higher at the beginning of the intervention and decreased gradually during the year.

^*∗*^2-3 sessions a week for 3-4 months.

**Table 2:** Sociodemographic characteristics of the *BeweegKuur* intervention participants.

	*N*	Study population
Age (years), mean (SD)	511	58.2 (10.9)
Sex (%)		
Male	210	40.8
Female	305	59.2
Civil status (%)		
Married	368	73.5
Living together	29	5.8
Divorced	24	4.8
Widow/widower	25	5.0
Single	55	11.0
Education (%)		
Lower education	123	39.5
Intermediate education	138	44.4
High education	50	16.1
Smoking behaviour (%)		
Smoker	28	13.6
Nonsmoker	178	86.4

Total number of participants is not similar for all sociodemographic characteristics as complete data are not available for all participants.

**Table 3:** Baseline measurements and changes in anthropometric outcomes and physical activity after one year.

	*N*	Baseline^1^	Change^1^
Weight (kg)	515	95.6 (94.0; 97.2)	−2.9 (−3.3; −2.5)^*∗∗∗*^
BMI (kg/m^2^)	515	33.0 (32.5; 33.5)	−1.0 (−1.2; −0.9)^*∗∗∗*^
Waist circumference (cm)	395	110.4 (109.1; 111.7)	−4.3 (−4.9; −3.7)^*∗∗∗*^
Blood glucose (mmol/L)	257	7.5 (7.2; 7.7)	−0.5 (−0.7; −0.3)^*∗∗∗*^
Systolic blood pressure (mmHg)	434	138.8 (137.3; 140.2)	−3.3 (−4.8; −1.9)^*∗∗∗*^
Diastolic blood pressure (mmHg)	432	82.4 (81.6; 83.3)	−2.6 (−3.4; −1.7)^*∗∗∗*^
Light to moderate physical activity (hours/week)	395	13.6 (12.3; 14.9)	2.1 (1.0; 3.2)^*∗∗∗*^
Vigorous physical activity (hours/week)	251	4.3 (3.5; 5.0)	1.7 (0.8; 2.5)^*∗∗∗*^

^1^Data are mean (95% confidence interval). ^*∗∗∗*^Statistical significant difference, paired sample *t*-test (*p* < 0.001).

**Table 4:** Changes in weight and waist circumference after one year, comparison between different subgroups.

	Weight change			Waist circumference change		
	*N*	(kg)^1,2^		*N*	(cm)^1,2^	
Sociodemographic factors						
Sex						
Male	210	−3.1 (−3.7; −2.5)		172	−4.5 (−5.3; −3.6)	
Female	305	−2.8 (−3.4; −2.2)		223	−4.2 (−5.1; −3.3)	
Age (years)						
<55	188	−3.8 (−4.7; −3.0)^*∗∗*a^		137	−4.1 (−5.2; −3.1)	
55–65	187	−2.6 (−3.3; −1.8)		157	−4.4 (−5.5; −3.4)	
>65	136	−2.2 (2.7; −1.6)^a^		98	−4.4 (−5.7; −3.1)	
BMI at baseline (kg/m^2^)						
<30	174	−2.1 (−2.6; −1.5)^*∗∗∗*a^		133	−3.9 (−4.8; −2.9)^*∗*^	
30–35	199	−2.6 (−3.2; −1.9)^b^		154	−3.7 (−4.6; −2.7)^a^	
>35	144	−4.4 (−5.4; −3.4)^a,b^		108	−5.8 (−7.3; −4.3)^a^	
Education						
Lower education	123	−3.1 (−4.0; −2.3)		105	−5.2 (−6.7; −3.8)	
Intermediate Education	138	−3.1 (−3.9; −2.3)		119	−4.7 (−5.9; −3.5)	
High education	50	−3.2 (−4.5; −1.9)		39	−4.1 (−5.8; −2.4)	
Uptake of the program						
Exercise program						
(1) independent program	158	−2.6 (−3.4; −1.8)		123	−3.1 (−4.1; −2.2)^*∗*a^	
(2) start-up program	163	−3.3 (−4.0; −2.5)		124	−4.7 (−5.8; −3.6)	
(3) supervised program	166	−2.9 (−3.7; −2.1)		132	−5.1 (−6.4; −3.8)^a^	
Number of individual sessions with dietician						
1–4	239	−2.6 (−3.1; −2.1)		196	−4.1 (−5.0; −3.3)	
4 or more	159	−3.5 (−4.4; −2.5)		126	−5.1 (−6.4; −3.8)	
Attendance group education sessions						
no	75	−1.7 (−2.8; −0.7)		47	−1.4 (−2.8; 0.1)^*∗*^	
yes	150	−2.3 (−3.1; −1.6)		127	−3.5 (−4.7; −2.3)	
Number of sessions with LSA						
1–3	205	−2.4 (−3.0; −1.8)		156	−3.3 (−4.3; −2.4)^*∗*^	
4 or more	253	−3.1 (−3.8; −2.5)		201	−4.8 (−5.7; −3.9)	
Changes in physical activity						
Change light and moderate physical activity (hours)						
<0	137	−2.3 (−3.2; −1.5)		102	−3.9 (−4.9; −2.8)	
0–3.5	116	−2.8 (−3.7; −1.9)		89	−4.2 (−5.8; −2.7)	
>3.5	123	−3.2 (−4.1; −2.4)		113	−5.1 (−6.3; −3.9)	
Change vigorous physical activity (hours)						
<0	82	−2.4 (−3.7; −1.2)		58	−4.5 (−6.3; −2.7)	
0–2	87	−2.9 (−3.8; −2.0)		75	−4.4 (−5.9; −2.9)	
>2	70	−3.5 (−4.7; −2.4)		59	−5.2 (−7.1; −3.3)	

Total number of participants is not similar for each factor as complete data are not available for all participants.

^1^Data are mean (95% confidence interval).

^2^Welch ANOVA was used to test significance between the groups. Statistically significant difference between the subgroups ^*∗*^(*p* < 0.05) ^*∗∗*^(*p* < 0.01) ^*∗∗∗*^(*p* < 0.001). Games-Howell post hoc tests for main effects. Superscript letters (a and b) indicate pairs of means that differ significantly from one another (*p* < 0.05).

## Abbreviations Used

BMI: Body mass index
FIN-D2D: Finnish National Diabetes Prevention Program
GGT: Greater Green Triangle
GP: General practitioner
LSA: Lifestyle advisor
NISB: Netherlands Institute for Sport and Physical Activity
PA: Physical activity.
