# Supplementary material for: One-Year Results of the BeweegKuur Lifestyle Intervention Implemented in Dutch Primary Healthcare Settings
Source: Biomed Res Int. 2015 Aug 25;2015:484823. doi: 10.1155/2015/484823 (PMC4561871; doi:10.1155/2015/484823)
Supplement: Supplementary file 1 — Supplemental Table 1: shows the socio-demographic characteristics of the Dutch population and BeweegKuur intervention participants. Supplemental Table 2: shows baseline measurements and changes in anthropometric outcomes and physical activity after one year split by gender. [file 484823.f1.pdf]

Supplemental Material

Schutte, BAM; Haveman-Nies, A; Preller, L. One-year results of the *BeweegKuur* lifestyle intervention implemented in Dutch primary healthcare settings.

Supplemental Table 1 Socio-demographic characteristics of the Dutch population and *BeweegKuur* intervention participants

Supplemental Table 2 Baseline measurements and changes in anthropometric outcomes and physical activity after one year split by gender

Supplemental Table 1 Socio-demographic characteristics of the Dutch population and *BeweegKuur* intervention participants

	Age group (years)	Dutch population	*BeweegKuur* participants
**Age**			
Mean age	Whole population	40.3	58.2
% of population between age 20 and 80*	20-40	34.5	5.3
	40-65	49.6	64.0
	65-80	16.0	30.7
**Sex (%)**			
Females	40-70	49.8	59.2
**Education (%)**			
Low	45-75	38.1	39.5
Intermediate	45-75	36.4	44.4
High	45-75	25.5	16.1
**Civil status (%)**			
Married	40-70	68.4	73.5
Living together	40-70		5.8
Divorced	40-70	13.2	4.8
Widow/widower	40-70	3.4	5.0
Single	40-70	15.0	11.0
**Smokers (%)**	12+	25.6	13.6
	40 to 50	28.4	
	50 to 55	31.6	
	55 to 65	26.1	
	65 to 75	17.6	

Total number of participants is not similar for all socio-demographic characteristics as complete data are not available for all participants

* age of BeweegKuur participants was between 20 and 81. Three persons were 81, therefore percentages don't add up to 100.

Reference: Centraal Bureau voor de Statistiek, Voorburg/Heerlen. URL: www.cbs.nl.

Tables:

Age: Bevolking; Kerncijfers

Civil status: Bevolking; geslacht, leeftijd, burgerlijke staat en regio, 1 januari

Education level: Bevolking; hoogst behaald onderwijsniveau; geslacht, leeftijd en herkomst

Civil status: Bevolking; geslacht, leeftijd, burgerlijke staat en regio, 1 januari

Smoking: Leefstijl, preventief onderzoek; persoonskenmerken

Supplemental Table 2 Baseline measurements and changes in anthropometric outcomes and physical activity after one year split by gender

	Males					Females				
	N	Baseline[1]		Change[1]		N	Baseline[1]		Change[1]	
Weight (kg)	210	101.3	(98.8;103.7)	-3.1	(-3.7;-2.5)***	305	91.7	(89.7;93.7)	-2.8	(-3.4;-2.2)***
BMI (kg/m^2)	210	31.9	(31.2;32.6)	-1.0	(-1.2;-0.8)***	305	33.8	(33.1;34.4)	-1.0	(-1.2;-0.8)***
Waist circumference (cm)	172	113.5	(111.6;115.5)	-4.5	(-5.3;-3.6)***	223	108.0	(106.3;109.7)	-4.2	(-5.1;-3.3)***
Blood glucose (mmol/l)	121	7.7	(7.3;8.1)	-0.6	(-1.0;-0.2)**	136	7.2	(7;7.5)	-0.5	(-0.7;-0.2)***
Systolic blood pressure (mmHg)	184	138.9	(136.7;141.0)	-2.2	(-4.5;0.1)	250	138.7	(136.7;140.6)	-4.2	(-6.1;-2.2)***
Diastolic blood pressure (mmHg)	184	82.5	(81.2;83.9)	-2.6	(-3.8;-1.3)***	248	82.4	(81.2;83.5)	-2.6	(-3.7;-1.4)***
Light to moderate physical activity (hours/week)	160	12.7	(10.6;14.8)	2.8	(1.1;4.4)*	235	14.2	(12.6;15.9)	1.6	(0.1;3.1)*
Vigorous physical activity (hours/week)	97	4.8	(3.4;6.2)	1.5	(0.1;2.8)*	154	3.9	(3.0;4.8)	1.8	(0.7;2.8)*

[1] Data are mean (95% Confidence Interval)
** Statistical significant difference, paired sample t-test ($p < 0.05$)
** Statistical significant difference, paired sample t-test ($p < 0.01$)
*** Statistical significant difference, paired sample t-test ($p < 0.001$)
